# Fermented Foods: Are They Tasty Medicines for *Helicobacter pylori* Associated Peptic Ulcer and Gastric Cancer?

**DOI:** 10.3389/fmicb.2016.01148

**Published:** 2016-07-25

**Authors:** Mydhily R. B. Nair, Deepak Chouhan, Sourav Sen Gupta, Santanu Chattopadhyay

**Affiliations:** Microbiome Research Facility, Microbiome Biology, Rajiv Gandhi Centre for BiotechnologyTrivandrum, India

**Keywords:** fermented food, probiotics, prevention of *H. pylori* infection, peptic ulcer, gastric cancer

## Abstract

More than a million people die every year due to gastric cancer and peptic ulcer. *Helicobacter pylori* infection in stomach is the most important reason for these diseases. Interestingly, only 10–20% of the *H. pylori* infected individuals suffer from these gastric diseases and rest of the infected individuals remain asymptomatic. The genotypes of *H. pylori*, host genetic background, lifestyle including smoking and diet may determine clinical outcomes. People from different geographical regions have different food habits, which also include several unique fermented products of plant and animal origins. When consumed raw, the fermented foods bring in fresh inocula of microbes to gastrointestinal tract and several strains of these microbes, like *Lactobacillus* and *Saccharomyces* are known probiotics. *In vitro* and *in vivo* experiments as well as clinical trials suggest that several probiotics have anti-*H. pylori* effects. Here we discuss the possibility of using natural probiotics present in traditional fermented food and beverages to obtain protection against *H. pylori* induced gastric diseases.

## Introduction

“Let food be thy medicine and medicine be thy food.”

Hippocrates (460 – 370 BC)

Every year peptic ulcer and gastric cancer takes approximately 301,000 and 740,000 lives, respectively (Piazuelo and Correa, 2013; Naghavi et al., 2015). Although both diseases have multiple etiologies like stress, diet, smoking and host genetic background, *Helicobacter pylori* infection is perhaps the most critical among them (Malfertheiner et al., 2014). However, every *H. pylori* infected individual does not develop peptic ulcer or gastric cancer. More than half of the world population is infected by *H. pylori*, but 10–20% of the infected people suffer from these diseases (Dorer et al., 2009). Why ∼80% of the *H. pylori* infected people in any given population never suffer from gastric disorders is unknown at present. Also, the clinical outcomes among the *H. pylori* infected population suffering from gastric disorders vary tremendously with geography (Covacci et al., 1999). For example, gastric cancer is fairly common in East-Asian countries like Japan and Korea, but in most African countries and India, the incidence of gastric cancer is low in spite of having high prevalence of *H. pylori* infection (Holcombe, 1992; Singh and Ghoshal, 2006; Shiota et al., 2013). Variations in bacterial and human genetic factors have been linked to explain the differences in clinical outcome, but our understanding of *H. pylori* infection and related diseases are really incomplete.

Microbiota is the ecological community of commensal, symbiotic and pathogenic microorganisms that literally share our body space. Microbiome is the combined genomes of the microbiota (Lederberg, 2001). Recent metagenomic analyses of DNA isolated from gastric tissue specimens show that human stomach is the niche of many bacterial species (Maldonado-Contreras et al., 2011). While the exact significance of the microbes that co-exist in highly acidic gastric milieu is not understood till date, it seems apparent that *H. pylori* infection can alter the dynamics of gastric microbiota (Andersson et al., 2008). However, microbiota can also be modulated by several other factors like alteration in immunity due to other infections and change in lifestyle including food and beverage consumptions (De Filippo et al., 2010). Interestingly, almost every geographical location has unique tradition of consuming fermented foods and beverages (Campbell-Platt, 1987). These fermented foods are rich source of bacteria, yeasts and molds and many of these microbes provide benefits to hosts and act as probiotics (Tamang et al., 2016a,b). More intriguingly, adding purified probiotics to therapy against *H. pylori* gives better eradication rate and reduces the side effects of antibiotics (Zhang et al., 2015). Unfortunately, however, the significances of the natural probiotics in traditional fermented foods and beverages are less studied in the context of *H. pylori* associated diseases. In this mini-review, we will discuss how probiotics present in different fermented foods and beverages may have a role in preventing *H. pylori* related gastric diseases.

## *H. pylori* Infection and Gastric Diseases

Presence of spiral bacilli in stomach have been reported several times during the past century, but the culture of this slow growing species remained unsuccessful until a serendipitous prolonged incubation of human gastric specimens in microaerophillic conditions during Easter holidays by Barry James Marshall and John Robin Warren (Doenges, 1938; Freedberg and Barron, 1940; Warren and Marshall, 1983; Marshall and Warren, 1984). To prove Koch’s postulate Barry Marshall drank pure culture of *H. pylori*, which resulted in hypochlorhydric vomiting and gastritis before he was treated with antibiotics (Marshall et al., 1985). Subsequently, a huge number of studies confirmed the role of *H. pylori* virulence factors in peptic ulcer and gastric cancer and *H. pylori* was classified as a type I carcinogen by WHO (Malfertheiner et al., 2014).

*H. pylori* expresses many virulence factors, but two multitasking proteins, the vacuolating cytotoxin (VacA) and the cytotoxin-associated gene A (CagA), seem to play the most crucial role in developing the gastro-duodenal diseases. The VacA is a secreted toxin, which forms large cytoplasmic vacuoles inside the host cells (Leunk et al., 1988). The VacA is also involved in reducing mitochondrial transmembrane potential, releasing cytochrome c, inducing cell death, activating MAP-kinases and inhibiting T-cell activation (Galmiche et al., 2000; Gebert et al., 2003; Willhite and Blanke, 2004; Yamasaki et al., 2006; Torres et al., 2007). The *vacA* gene has mosaic structures viz. s1/s2 alleles (encoding signal peptides), m1/m2 alleles (encoding mid-regions) and i1/i2/i3 (encoding intermediate regions) (Cover et al., 1994; Atherton et al., 1995, 1999; Rhead et al., 2007). The s1 and the i1 alleles of *vacA* are associated with aggressive clinical outcomes (Rhead et al., 2007; Yamaoka, 2010). The *H. pylori* strains carrying *vacA* s1 usually carry *cagA* gene, which is located in the *cag-*pathogenicity island (Blaser et al., 1995; Xiang et al., 1995; Yamaoka, 2010). The *cagA^+^* strains are associated with more severe diseases in most regions (Blaser et al., 1995). The *cagA* gene shows length polymorphism at the 3′ end and this variable region encodes EPIYA motifs that undergo phosphorylation once the CagA protein is translocated into the host cells (Yamaoka et al., 1998; Higashi et al., 2002). The phospho-CagA interacts and deregulates the SHP-2 protein, which leads to cancer, but the CagA can hijack cellular pathways also by phosphorylation independent manner (Higashi et al., 2002; Hatakeyama, 2014).

Polymorphisms in host immune genes also contribute to determine the clinical status of the host (Datta De and Roychoudhury, 2015). For example, polymorphisms in interleukin-1 (IL1) and tumour necrosis factor (TNF) genes have been shown to play important roles in progression of gastric diseases among Scottish, Japanese, American, and Indian populations (El-Omar, 2001; Datta De and Roychoudhury, 2015). Moreover, every geographic region has unique lifestyle including food and beverage intakes, which are known to have effects on gut microbiota (De Filippo et al., 2010).

It is now appreciated that human stomach microbiota consists of 44 bacterial phyla, dominated by four phyla: Proteobacteria, Firmicutes, Actinobacteria, and Bacteroidetes (Maldonado-Contreras et al., 2011). A study using Swedish patients showed that the presence of *H. pylori* in stomach may significantly alter the relative abundance of other bacteria (Andersson et al., 2008). Colonization by specific groups of bacteria seems to correlate with *H. pylori* infection status. *H. pylori* colonization dramatically reduced the diversity and increased the colonization of Proteobacteria. Positive *H. pylori* status in America is also associated with increased abundance of Proteobacteria, Spirochetes and Acidobacteria, and with decreased abundance of Actinobacteria, Bacteroidetes and Firmicutes (Maldonado-Contreras et al., 2011). Recently, in mouse, it has been shown that *H. pylori* colonization can influence both gastric and intestinal microbiota (Kienesberger et al., 2016). While it appears that the stomach and intestinal microbiota in the presence and in the absence of *H. pylori* infection may have a role in gastric diseases the mechanism is not known.

Treatment for all *H. pylori* infections has been recommended for several geographical locations (Shiota et al., 2013; Malnick et al., 2014). The usual treatment regimen for *H. pylori* is a short course of two antibiotics (mostly clarithromycin and amoxicillin) along with proton pump inhibitors (e.g., omeprazole or lansoprazole). The treatment, however, is complicated by several factors like bacterial resistance to antibiotics, re-infection, side effects (bloating, diarrhea and taste disturbances) and alteration of healthy gut microbiota (Malnick et al., 2014; Zhang et al., 2015). The destruction of the commensal flora may lead to increased prevalence of opportunistic pathobionts, like *Clostridium difficile* (Malnick et al., 2014). Hence, the treatment of *H. pylori* using antibiotics has the risk for microbiota imbalance or dysbiosis, which may lead to other diseases. Also, eradication of *H. pylori* may lead to esophageal cancer (Blaser, 2008; Blaser and Falkow, 2009). Therefore, alternative approach that can eradicate or prevent *H. pylori* infection without affecting gut microbiota is needed.

## Use of Probiotics for the Eradication of *H. pylori*

Probiotics (means ‘for life’) are live microorganisms which provide beneficial effects when taken in sufficient quantity. Examples include several species of *Lactobacillus, Bifidobacterium, Enterococcus, Lactococcus, Streptococcus* as well as *Saccharomyces* (Reid, 1999; Fijan, 2014). Probiotics are known to have beneficial roles in curing antibiotic associated diarrhea, constipation, traveler’s diarrhea, food allergies and cancer (McFarland, 2007; Chmielewska and Szajewska, 2010; Hempel et al., 2012; Isolauri et al., 2012; Russo et al., 2014).

*Lactobacillus* is normally present in human intestinal tract including stomach and it is tolerant to acid and bile (Ruiz et al., 2013). Therefore, *Lactobacillus* is an attractive candidate for probiotic for the treatment of *H. pylori* related gastric diseases. Bhatia et al. (1989) showed that the culture supernatant of *Lactobacillus acidophilus* inhibits *H. pylori in vitro* due to an extracellular secretory product. Direct application of *L. acidophilus* on blood agar plate can also inhibit *H. pylori* (Vilaichone et al., 2002). Subsequently, it was found that both *L. acidophilus* and *L. casei* subsp. *rhamnosus* can inhibit *H. pylori* due to the production of lactic acid (Midolo et al., 1995; Enany and Abdalla, 2015). The lactic acid produced by *L. casei* strain Shirota inhibits 70% of urease activity *in vitro* and significantly reduces the levels of *H. pylori* colonization in mouse model (Sgouras et al., 2004). Lorca et al. (2001) studied antibacterial activity of 17 *Lactobacillus* strains on 10 *H. pylori* strains and concluded that the inhibition was due to acid production. They also found that autolysis of *L. acidophilus* after 24 h of culture releases a proteinaceous compound and this event is related to the bactericidal effect (Lorca et al., 2001). Furthermore, *H. pylori* colonized mice when treated with a commercial mixture of live probiotics (*L. rhamnosus*, strain R0011, and *L. acidophilus*, strain R0052) they suppressed colonization of *H. pylori* strain SS1 (Johnson-Henry et al., 2004).

The sulfatide-binding protein of the *L. reuteri* competes and binds to the gangliotetraosylceramide (asialo-GM1) and sulfatide, which are putative receptors of *H. pylori* (Mukai et al., 2002). *Weissella confusa* can inhibit *H. pylori* adherence to human gastric cell line by 90%. (Nam et al., 2002).

*H. pylori* infected MKN45 cells showed increased expression of Smad7 and NFkB, and induced pro-inflammatory cytokines IL-8 and TNF-α *in vitro*. Probiotic *L. acidophilus* pre-treatment, however, inactivate the Smad7 and NFkB pathways and reduces the *H. pylori* induced inflammation (Yang et al., 2012). Using gnotobiotic murine model, it was shown that *L. salivarius* infection also inhibits the colonization of *H. pylori* and associated inflammatory responses like IL-8 release (Kabir et al., 1997; Avía et al., 1998).

Since *in vitro* experiments and *in vivo* mouse studies showed promising results, a significant number of clinical trials have been performed in the recent past (**Table 1**). Several meta-analyses published in 2013 revealed that addition of probiotics in triple therapy against *H. pylori* improves overall efficacy and reduces the side effects of therapy like nausea, diarrhea metallic taste, abdominal/epigastric pain (Ruggiero, 2014). However, it needs further improvement since the benefits conferred by the probiotics are often not too remarkable. For example, a meta-analysis based on literature search strategy suggest that use of probiotics (mostly *Lactobacillus, Bifidobacterium* and *Streptococcus* and in few trials *Enterococcus, Clostridium, Saccharomyces* etc) in triple therapy improve eradication rate of *H. pylori* by ∼10% and reduce adverse effects of therapy by ∼15% (Zhang et al., 2015).

**Table 1:** Some of the anti-*Helicobacter pylori* clinical trials and meta-analyses that used probiotics.

Study	Species	Results	Reference
Meta-analysis	*Lactobacillus* strains	Improvement in eradication rates	Zheng et al., 2013
Randomized open label clinical study	*Bifidus infantis*	Used as adjuvant improves cure rate	Dajani et al., 2013
Meta-analysis	*Lactobacillus* and *Bifidobacterium* species	Beneficial effects on eradication rate and incidence of side effect	Wang et al., 2013
Meta-analysis	*Lactobacillus acidophilus, Lactobacillus casei* DN-114001, *Lactobacillus gasseri, Bifidobacterium infantis* 2036.	Increases eradication rates	Dang et al., 2014
Clinical trials	*Lactobacillus gasseri* OLL2716(LG21)	Supression of *H. pylori*, reduction in gastric mucosal inflammation	Sakamoto et al., 2001
Double blind randomized placebo-controlled crossover clinical study	*Lactobacillus reuteri* strain SD2112	Suppression of urease activity and *H. pylori* density	Imase et al., 2007
Double blind placebo-controlled study	*Lactobacillus reuteri* ATCC 55730	Suppresses *H. pylori* infection, decreases the occurrence of dyspeptic symptoms	Francavilla et al., 2008
Double blind placebo-controlled study	*Lactobacillus reuteri* ATCC 55730	*H. pylori* eradicated in half of the patients by omeprazole plus *L. reuteri*	Saggioro et al., 2005
Double blind randomized placebo-controlled study	*Lactobacillus reuteri* DSM 17938, *Lactobacillus reuteri* ATCC PTA 6475	Combination of both strains alone exert an inhibitory effect and when used with eradication therapy reduces side effects	Francavilla et al., 2014
Open label single center study	*Lactobacillus reuteri* DSM 17938	Reduction of urease activity	Dore et al., 2014
Single center, double-blind, prospective, randomized, placebo-controlled trial	*Lactobacillus GG*	Reduced side effects and overall treatment tolerability	Armuzzi et al., 2001

## Role of Fermented Foods and Beverages Against *H. pylori* Associated Diseases

Fermentation of food dates back to the early ages of human evolution and provides an effective way of preserving food for longer durations (McGovern et al., 2004). Many of the bacteria, yeasts and molds that are present in fermented foods and beverages are known probiotics and probably provide health benefits when consumed raw (Stanton et al., 2005). The significance of the microbes present in fermented food in maintaining human health was first noticed by Elie Metchnikoff (Mackowiak, 2013). He hypothesized that the long and healthy lives of Bulgarian peasants were due to the regular consumption of sour milk and yogurt containing the necessary beneficial microbes (Mackowiak, 2013).

Many of the probiotic that are isolated directly from the fermented foods, particularly fermented dairy products, have anti *H. pylori* effects. Based on dietary interviews it was found that yogurt, but not unfermented dairy products, when consumed one serving per week or more has protective effect against *H. pylori* infection in Mexican population (Ornelas et al., 2007). Several strains of *Lactobacilli* and two strains of yeast directly isolated from yogurt were found to have inhibitory effect on *H. pylori* (Oh et al., 2002). A meta-analysis of randomized controlled trials shows that there is ∼10% improvement in eradication rates when using fermented milk based probiotics, which seems to be better than capsule/sachet-based bacteria-only preparations (Sachdeva and Nagpal, 2009). Similarly, 4-week treatment with *L. gasseri*-containing yogurt improved the efficacy of triple therapy in patients with *H. pylori* infection (Deguchi et al., 2012). Another study showed that *H. pylori* infected children have a lower number of *Bifidobacterium* in their gut, but intake of probiotics-containing yogurt had multiple effects like, restoration of *Bifidobacterium*, reduction of *H. pylori* load, increase in IgA and decrease in IL-6 (Yang and Sheu, 2012). Three strains of lactic acid bacteria, LY1, LY5 AND IF22, which are from the spent culture supernatant of fermented milk, showed anti-*H. pylori* effect (Lin et al., 2011). In china, several probiotics from traditional fermented foods were isolated and two strains of *Lactobacillus- L. plantarum 18* and *L. gasseri* showed potential anti-*H. pylori* activity (Chen et al., 2010). Kefir, a fermented milk product was found to be effective in eradication and reducing side effects when used along with triple therapy (Bekar et al., 2011). An *in vitro* study proved that *L. plantarum* (MLBPL1) isolated from sauerkraut (fermented cabbage) had an anti-*Helicobacter* activity (Rokka et al., 2006). Interestingly, the main inhibitory activity is mostly associated with cell wall.

Unfortunately, however, anti-*H. pylori* activity alone does not ensure protection from gastric diseases and gastric cancer may sometimes develop even after eradication of *H. pylori* since some of the *H. pylori* proteins like CagA may act by ‘hit and run’ mechanism (Shiota et al., 2013; Hatakeyama, 2014). More interestingly, prevalence of *H. pylori* and incidence of gastric diseases does not match in some countries. In Africa and India, the prevalence of *H. pylori* infection and associated gastritis is high, but the incidence of gastric cancer is very low (Holcombe, 1992; Singh and Ghoshal, 2006). On the other hand, East-Asian countries like Japan and Korea have high rates of gastric cancer (Singh and Ghoshal, 2006). Genotype alone cannot be responsible to explain the clinical outcome since nearly all *H. pylori* strains isolated from East-Asia are virulent (Shiota et al., 2013). Therefore, it is intriguing to compare the microbes that are present in traditional fermented foods and beverages of Japan or Korea and African countries (**Table 2**). Apparently, fermented foods in African countries are based on milk, beans, grains and roots. They are dominated by *Lactobacillus* and other lactic acid bacteria. Conversely, Japanese fermented foods are primarily based on rice, soy and fish and these foods have varieties of bacteria and fungi. Interestingly, the soy foods may reduce the risk for gastric cancer, while high salt containing foods might be a risk factor in Japan and Korea (Hirayama, 1981; Woo et al., 2013).

**Table 2:** Microbes present in traditional fermented foods and beverages in Japan and Africa.

Fermented food	Ingredients	Microorganism	Known probiotics or anti-*H. pylori* activity	Country	Reference
**Fermented food of Japan and Korea**					
Sake	Rice	*Aspergillus sojae, Bacillus subtilis* and lactic acid bacteria	Lactic acid bacteria and *Bacillus subtilis*	Japan	Sakaguchi, 1958a,b
Narezushi	Fish, salt and cooked rice	*L. plantarum* and *L. brevis*	*L. plantarum*	Japan	Kiyohara et al., 2012
Takju	Rice	*Lb. harbinensis, Lb. parabuchneri Lactobacillus (Lb.) paracasei, Lb. plantarum*, and *Leuconostoc pseudomesenteroides*	*L. plantarum*	Korea	Kim et al., 2010
Vinegar	Rice	*Aspergillus oryzae, Lactobacillus acetotolerance, Acetobacter pasteurianus, Saccharomyces* sp. and lactic acid bacteria	Lactic acid bacteria and *Saccharomyces* sp.	Japan	Haruta et al., 2006
Natto	Soybean	*Bacillus subtilis*	*Bacillus subtilis*	Japan	Kubo et al., 2011
Starch Noodle	Starch from sweet potato, mung bean etc	*L. casei, L. cellobiosus, L. fermenti*	*L. casei*	Korea, Japan	Rhee et al., 2011
Kimchi	Korean cabbage, radish, various vegetables, salt	*L. mesenteroides, L. brevis, L. plantarum*	*L. plantarum*	Korea	Rhee et al., 2011
Miso	Soybean and sometime rice or barley	*Aspergillus oryzae Saccharomyces cerevisiae and lactic acid bacteria*	Lactic acid bacteria and *Saccharomyces* sp.	Japan	Hirayama, 1981
Komesu and kurosu	Rice	*Aspergillus oryzae, Saccharomyces cerevisiae* and acetic acid bacteria	*Saccharomyces* sp.	Japan	Nanda et al., 2001
Tempeh	Soybean	*Rhizopus* sp.	?	Japan	Aoki et al., 2003
**Fermented food of Africa**					
Rigouta	Milk	*Lactococcus lactis and Enterococcus faecalis*	*Enterococcus faecalis*	Tunisia	Ghrairi et al., 2004
Wara	Cow milk	*Lactobacillus plantarum* and other lactic acid bacteria	*Lactobacillus plantarum* and other lactic acid bacteria	Nigeria	Olasupo et al., 1997
Ugba	Oil bean seed	*Bacillus subtilis*	*Bacillus subtilis*	Nigeria	Olasupo et al., 1997
Fufu	Cassava	*Lactobacillus plantarum* and other lactic acid bacteria	*Lactobacillus plantarum* and other lactic acid bacteria	Nigeria	Olasupo et al., 1997
Ogi	Maize	*Lactobacillus plantarum* and other lactic acid bacteria	*Lactobacillus plantarum* and other lactic acid bacteria	Nigeria	Olasupo et al., 1997
Kunu-zarki	Millet	*Lactobacillus plantarum* and other lactic acid bacteria	*Lactobacillus plantarum* and other lactic acid bacteria	Nigeria	Olasupo et al., 1997
Kenkey	Maize	*Lactobacillus plantarum* and other lactic acid bacteria	*Lactobacillus plantarum* and other lactic acid bacteria	Nigeria	Olasupo et al., 1997
Iru	African locust bean	*Lactobacillus plantarum* and other lactic acid bacteria	*Lactobacillus plantarum* and other lactic acid bacteria	Nigeria	Olasupo et al., 1997
Garri	Cassava	Yeast, *Lactobacillus plantarum, Leuconostoc fallax, Lactobacillus fermentum* and other lactic acid bacteria	*Lactobacillus plantarum* and other lactic acid bacteria	Nigeria and other part of Africa	Kostinek et al., 2005
Kule naoto	Milk	*Lactobacillus plantarum* and other lactic acid bacteria	*Lactobacillus plantarum* and other lactic acid bacteria	Maasai in Kenya	Mathara et al., 2004
Poto Poto	Maize dough	*Lactobacillus plantarum, Bacillus* sp., *Lactobacillus reuteri, Lactobacillus casei* and other lactic acid bacteria	*Lactobacillus plantarum, Lactobacillus reuteri, Lactobacillus casei*	Congo	Abriouel et al., 2006
Degue	Pearl millet dough	*Lactobacillus plantarum, Bacillus* sp., *Lactobacillus reuteri, Lactobacillus casei*, other lactic acid bacteria and yeast and molds	*Lactobacillus plantarum, Lactobacillus reuteri, Lactobacillus casei*	Burkina Faso	Abriouel et al., 2006

A similar comparison would be exiting between the fermented foods of ethnic populations in North-Eastern India (e.g., ethnic populations in Sikkim state like Bhutias) and Mid-Eastern India (e.g., ethnic population of Jharkhand and West Bengal states like Santhals). North-Eastern states have highest incidence of gastric cancer in India (Pradhan et al., 2003–2004). This high prevalence has been thought to be due to smoking and high salt consumption that possibly come from fermented and pickled foods including fish and meat (Phukan et al., 2005; Verma et al., 2012). Recent analyses of some of the fermented foods showed presence of huge microbial variety but their significances in gastric diseases have not been studied (Tamang and Sarkar, 1996; Tamang et al., 2016a,b). Unfortunately, not much is known about the microbes that are present in fermented foods consumed by the Santhals. But interestingly, they regularly consume intoxicating alcoholic beverages like Handia and Mahua fermented in traditional way and these beverages are not common elsewhere (Kumar and Rao, 2007). Among Santhals, infections with virulent *H. pylori* strains are extremely common without any manifestation of gastric diseases (Datta et al., 2003).

Our current understandings of microbes present in the ethnic fermented foods are incomplete at present, but with modern methodologies like metagenomic analysis using Next-generation sequencing the microbial species are now easy to identify (Mozzi et al., 2013). However, to prove or disprove the hypothesis—whether or not microbes present in the ethnic fermented food can protect certain population from peptic ulcer or gastric cancer is very tricky, particularly when *H. pylori* infection is not the only determinant in precipitating the gastric diseases (Parekh et al., 2014; De and Roychoudhury, 2015). How the microbes present in the ethnic fermented food can alter the pathogenicity of *H. pylori* in combination with gastric and duodenal microbiome as well as host immunity for different population is perhaps the key question at present.

## Conclusion

*H. pylori* infection is the major risk factor for peptic ulcer and gastric cancer and the eradication of this bacterium using antibiotics is often unsuccessful. Several microbes with known probiotic activities are shown to have inhibitory effects against *H. pylori in vitro* and *in vivo*. Inclusion of probiotics in triple therapy leads to improved efficacy and reduced side effects. Most traditional fermented foods and beverages are natural sources of probiotic microbes. Microbes directly isolated from the fermented products are shown to have anti-*H. pylori* activity. Few studies showed that consumption of probiotics containing yogurt and kefir are somewhat beneficial in the context of *H. pylori* infection. Many ethnic populations have significantly low incidences of peptic ulcer and gastric cancer in spite of having very high prevalence of *H. pylori* infection. Incidentally, each ethnic population also has unique tradition of consuming fermented food and beverages that contain probiotics. It is intriguing to hypothesize that regular consumptions of these probiotics may have protective effect against peptic ulcer and gastric cancer for some populations. Analyzing these traditional fermented foods and beverages using modern techniques is needed to understand these microbes and their significances.

## Author Contributions

MN and DC equally contributed 60% of the mini-review works. SG contributed 15% and SC contributed 25% in the mini-review works.

## Conflict of Interest Statement

The authors declare that the research was conducted in the absence of any commercial or financial relationships that could be construed as a potential conflict of interest.
